# The First Insight into Polyhydroxyalkanoates Accumulation in Multi-Extremophilic *Rubrobacter xylanophilus* and *Rubrobacter spartanus*

**DOI:** 10.3390/microorganisms9050909

**Published:** 2021-04-24

**Authors:** Xenie Kouřilová, Jana Schwarzerová, Iva Pernicová, Karel Sedlář, Kateřina Mrázová, Vladislav Krzyžánek, Jana Nebesářová, Stanislav Obruča

**Affiliations:** 1 Department of Food Chemistry and Biotechnology, Faculty of Chemistry, Brno University of Technology, Purkynova 118, 612 00 Brno, Czech Republic; xckourilovax@fch.vut.cz (X.K.); xcpernicovai@fch.vut.cz (I.P.); 2 Department of Biomedical Engineering, Faculty of Electrical Engineering and Communication, Brno University of Technology, Technicka 12, 616 00 Brno, Czech Republic; xschwa16@stud.feec.vutbr.cz (J.S.); sedlar@feec.vutbr.cz (K.S.); 3 Institute of Scientific Instruments of the Czech Academy of Sciences, v.v.i., Kralovopolska 147, 612 64 Brno, Czech Republic; mrazova@isibrno.cz (K.M.); krzyzanek@ISIBrno.Cz (V.K.); 4 Biology Centre, The Czech Academy of Sciences, v.v.i., Branisovska 31, 370 05 Ceske Budejovice, Czech Republic; nebe@paru.cas.cz; 5 Faculty of Science, Charles University, Vinicna 7, 128 44 Prague 2, Czech Republic

**Keywords:** *Rubrobacter xylanophilus*, *Rubrobacter spartanus*, polyhydroxyalkanoates, stress conditions, extremophiles

## Abstract

Actinobacteria belonging to the genus *Rubrobacter* are known for their multi-extremophilic growth conditions—they are highly radiation-resistant, halotolerant, thermotolerant or even thermophilic. This work demonstrates that the members of the genus are capable of accumulating polyhydroxyalkanoates (PHA) since PHA-related genes are widely distributed among *Rubrobacter* spp. whose complete genome sequences are available in public databases. Interestingly, all *Rubrobacter* strains possess both class I and class III synthases (PhaC). We have experimentally investigated the PHA accumulation in two thermophilic species, *R. xylanophilus* and *R. spartanus*. The PHA content in both strains reached up to 50% of the cell dry mass, both bacteria were able to accumulate PHA consisting of 3-hydroxybutyrate and 3-hydroxyvalerate monomeric units, none other monomers were incorporated into the polymer chain. The capability of PHA accumulation likely contributes to the multi-extremophilic characteristics since it is known that PHA substantially enhances the stress robustness of bacteria. Hence, PHA can be considered as extremolytes enabling adaptation to extreme conditions. Furthermore, due to the high PHA content in biomass, a wide range of utilizable substrates, Gram-stain positivity, and thermophilic features, the *Rubrobacter* species, in particular *Rubrobacter xylanophilus*, could be also interesting candidates for industrial production of PHA within the concept of Next-Generation Industrial Biotechnology.

## 1. Introduction

The genus *Rubrobacter* accommodates non-motile actinobacteria which are obligatory aerobic, asporogenic and Gram-stain positive [1]. A very important feature of the genus is its ability to survive under various stress conditions. Numerous strains belonging to the genus are highly resistant to UV and even ionogenic radiation, actually, they can rival the canonical radiation-resistant species of the genus *Deinococcus* in this regard [2]. Apart from irradiation resistance, members of the genus also commonly exhibit halotolerant and thermophilic attributes. The genus *Rubrobacter* is currently divided into two main clusters: one cluster comprises the thermophilic species, such as *R. xylanophilus*, *R. calidiflumis* and *R. naidicus* with an optimal growth temperature of about 60 °C; and the second cluster includes the thermophilic species, *R. taiwanensis* and *R. spartanus* with optimal growth above 50–60 °C along with the slightly thermophilic species *R. radiotolerans*, which grows best at 46–48 °C and also the mesophilic species, such as *R. bracarensis*, *R. aplysinae* or *R. marinus* [3,4].

Due to their multi-extremophilic features, species of the *Rubrobacter* genus are of fundamental importance for the understanding of various stress adaptation strategies. It has been reported that radiotolerance of *Rubrobacter* spp. is a result of multiple protective mechanisms. The specific pigments deinoxanthin and bacterioruberin serve as antioxidants protecting bacterial cells from radicals induced by radiation. Moreover, *Rubrobacter* species also possess the potent superoxide dismutase, an enzyme capable of efficient radical elimination [5] and also an enzyme machinery enabling the repair of radiation-damaged DNA [6].

Apart from the fundamental importance of understanding adaptation mechanisms to stress conditions and exploring the limits of life, extremophiles are also of interest to the biotechnological industry. At first, they are capable of the production of extremozymes, i.e., robust and stable enzymes that find applications in various fields of biotechnology. *R. xylanophilus* was identified as a source of a highly stable D-amino acid oxidase [7] and also a highly alkalophilic phenylalanine ammonia-lyase [8]. Furthermore, extremolytes, i.e., metabolites providing a stress-protective function, find numerous applications in the food industry, cosmetics or medicine [9]. Members of the *Rubrobacter* genus are capable of producing the valuable pigment bacterioruberin [6], compatible solutes [10], or extraordinary high amounts of glutathione [11]. Besides the production of extremozymes and extremolytes, extremophilic microorganisms can be also employed in Next-Generation Industrial Biotechnology (NGIB). This concept was recently suggested by Prof. Chen and emphasizes the insensitivity of extremophile-based biotechnological processes against microbial contamination, which might have very positive consequences on the suitability and feasibility of the biotechnological processes [12].

Polyhydroxyalkanoates (PHA) are microbial polyesters that are accumulated in the form of intracellular inclusions by numerous prokaryotic species. The homopolymer of 3-hydroxybutyrate, poly(3-hydroxybutyrate) (P3HB), is the most widespread and best-studied member of the PHA family. Actually, due to their technological and mechanical properties, biodegradability, biocompatibility, and renewable origin, PHA are considered to be a “green” alternative to petrochemical “plastics” [13]. From a biological point of view, the main purpose of PHA in bacterial cells is the storage of carbon and energy. Furthermore, it has been recently reported that the presence of PHA granules, as well as a complex PHA metabolism, can also enhance the robustness and resistance of bacterial cells to numerous stress factors, such as fluctuations in osmotic pressure [14], heating [15], freezing-thawing [16], UV irradiation [17], or oxidative pressure [18]. Actually, several very recent reviews are dedicated to the stress-protective function of PHA for prokaryotic cells [19,20].

Using BLAST analysis, we have identified the presence of genes encoding for PHA synthetic machinery in several members of the *Rubrobacter* genus whose genome sequences were already published and are available in public databases. We have also performed a basic bioinformatics analysis of the available genomes of the *Rubrobacter* spp with respect to the PHA metabolism. To the best of our knowledge, the PHA production has not been reported for any member of the *Rubrobacter* genus so far. Since *Rubrobacter* spp. are Gram-positive, non-sporulating, extremophilic bacteria with a wide range of utilizable substrates, which are biotechnologically very important features, the main aim of this study was to investigate the PHA synthetic capability and potential of the selected biotechnologically interesting *Rubrobacter* species—in particular *R. xylanphilis* and *R. spartanus*.

## 2. Materials and Methods

### 2.1. Microorganisms

Bacteria *Rubrobacter xylanophilus* DSM 9941 and *Rubrobacter spartanus* DSM 102139 were obtained from German public collection of microorganisms, Leibnitz Institute DSMZ-German Collection of Microorganisms and Cell Cultures, Braunschweig, Germany.

### 2.2. Cultivation and PHA Production

The tryptone soya broth CM0129 (Oxoid) complex medium was used for inoculum growth in most of the experiments. Compared with the manufacturer-recommended concentration (30 g/L), the appropriate concentration was 5 times lower (6 g/L). The *Thermus* medium [21] supplemented by yeast extract (2.5 g/L and 5 g/L) was also tested for inoculum development in the initial experiments. The inoculum was cultivated for 24 h under constant shaking of 180 rpm and then used for inoculation (10% (*v/v*) of the production medium). A mineral salt medium (MSM) containing Na_2_HPO_4_ · 12 H_2_O (9.0 g/L), KH_2_PO_4_ (1.5 g/L), NH_4_Cl (1.0 g/L), MgSO_4_ · 7 H_2_O (0.2 g/L), CaCl_2_ · 2 H_2_O (0.02 g/L), Fe^(III)^NH_4_citrate (0.0012 g/L), yeast extract (0.5 g/L), 1 mL/L trace element solution (EDTA (50.0 g/L), FeCl_3_ · 6 H_2_O (13.8 g/L), ZnCl_2_ (0.84 g/L), CuCl_2_ · 2 H_2_O (0.13 g/L), CoCl_2_ · 6 H_2_O (0.1 g/L), MnCl_2_ · 6 H_2_O (0.016 g/L), H_3_BO_3_ (0.1 g/L), dissolved in distilled water) and carbon source (usually 20 g/L, unless defined otherwise) was chosen for the PHA production. MSM, trace element solution and carbon substrate were sterilized separately. Further manipulation was aseptic. The cultivations were performed in 250 mL Erlenmeyer flasks containing 100 mL of cultivation media. All cultivations in MSM lasted 72 h under constant shaking (180 rpm). Then, the cultivations were terminated and the cells were subsequently analyzed as described below.

For the initial screening of the substrate utilization ability of the strains, the temperature of cultivation was set at 50 °C. The tested substrates were arabinose, fructose, galactose, glucose, glycerol, lactose, mannose, waste frying oil, sucrose, and xylose. Based on the results of the substrate screening, glucose (*R. xyalnophilus*) and glycerol (*R. spartanus*) were used as the preferred substrates for further experiments. The effect of temperature on the production was tested in the range of 45–60 °C. Finally, the ability to produce copolymers was also investigated. Valeric acid, levulinic acid, n-propanol, n-amyl alcohol, and sodium propionate were used as precursors for the incorporation of 3-hydroxyvalerate monomers. These precursors were added to the MSM with an appropriate carbon source (20 g/L glycerol in *R. spartanus*, 20 g/L glucose in *R. xylanophilus*) at a concentration of 2 g/L. 1,4-butanediol, 1,6-hexanediol, γ-butyrolactone, and ε-caprolactone were tested as structural precursors of 4-hydroxybutyrate. These compounds were the sole carbon source and were added to the medium at a concentration 8 g/L. Precursors leading to the production of medium-chain-length PHA (mcl-PHA) have also been tested. For this purpose, hexanoate and octanoate were used at a concentration of 2 g/L along with 20 g/L of glucose or glycerol for *R. xylanophilus* and *R. spartanus*, respectively.

### 2.3. Analyses

The obtained biomass was determined gravimetrically as the cell dry mass (CDM). For the analysis, parallel samples, each consisting of 10 mL of suspension, were taken from the cultivations. These samples were centrifuged for 5 min (6000× *g*), then washed with distilled water and centrifuged again. The biomass thus treated was dried to constant weight. A portion of the dry samples was taken to determine the content and monomer composition of PHA by gas chromatography with a flame ionization detector (GC-FID). The determination was performed according to a previously described method [22].

The morphology of both investigated cultures was assessed employing a Transmission Electron Microscopy (TEM) analysis using the microscope JEOL 1010 (JEOL, Pleasanton, CA, USA) as described previously [23]. The samples of both cultures were centrifuged and a concentrated pellet was pipetted on 3 mm aluminum carriers pretreated with 1% solution of lecithin in chloroform and fixed using the high-pressure freezer EM ICE (Leica Microsystems, Vienna, Austria). Frozen samples were transferred into the freeze substitution unit AFS2 (Leica Microsystems, Vienna, Austria). The substitution protocol described in [23] was modified. The substitution solution contained 1,5% OsO_4_ in acetone, the initial phase of the freeze-substitution was set to −90 °C for 72 h, followed by warming up to −20 °C for 24 h (5 °C per hour) and finished at 4 °C for 18 h (3 °C per hour). The samples were then washed 3 times for 15 min with acetone, gradually infiltrated with mixtures of epoxy resin (Epoxy Embedding Medium kit, Sigma-Aldrich) and acetone in the ratios 1:2, 1:1, 2:1 and pure resin (1 h each). The samples were then left in fresh pure resin overnight under vacuum and were finally embedded in fresh pure resin using 62 °C heat for 48 h. The embedded samples were then cut to ultrathin sections using a diamond knife with a cutting angle of 45° (Diatome, Nidau, Switzerland) and an ultramicrotome UTC Ultracut (Leica Microsystems, Vienna, Austria). The sections were stained with uranyl acetate and lead citrate.

### 2.4. Bioinformatics Analyses

Manual identification of the PHA synthases coding genes was done with BLAST [24] using available sequences in the GenBank [25] and Uniprot [26] databases. An operon structure was predicted with the Operon-mapper [27]. PHA depolymerases were identified using the PHA Depolymerase Engineering Database [28]. The molecular weight of the identified PHA synthase subunits was predicted with the sequence manipulation suite [29].

## 3. Results

### 3.1. PHA Synthases in Genus Rubrobacter

There are currently 46 assemblies of *Rubrobacter* genomes publicly available in the GenBank database. While 20 assemblies are low-quality unannotated genomes, there are also six complete genome sequences. All of these assemblies prove that bacteria of the genus *Rubrobacter* carry the machinery needed for PHA synthesis. While we manually identified putative genes coding Class I and Class III PHA synthases, see Table 1, we obtained an additional 43 hits for other phaC and phaE genes in the remaining draft but annotated *Rubrobacter* genomes (data not shown). We observed that the analyzed genomes not only contain Class I PHA synthases formed by a single unit coded by the phaC gene, but all complete genomes carry also a Class III PHA synthase that is formed by two subunits coded by phaC and phaE genes. In all the cases, genes coding two subunits of the Class III PHA synthase were found in a single operon.

Similarly, to PHA synthases, genomes of bacteria from the genus *Rubrobacter* carry also genes coding PHA depolymerases. We found 100 hits corresponding to the superfamily “Intracellular nPHAscl depolymerases (lipase box)” and 41 hits corresponding to the superfamily “Intracellular nPHAmcl depolymerases”. Surprisingly, 15 extracellular depolymerases belonging to the superfamily “Extracellular dPHAscl depolymerases (catalytic domain type 1)” were also found, see Table 2.

### 3.2. PHA Accumulation by R. spartanus and R. xylanophilus

Based on the BLAST analysis, it was supposed that *R. spartanus* and *R. xylanophilus* reveal the ability to produce and accumulate PHA. This assumption was also verified at the phenotype level. First, the optimal composition of the complex media for the inoculum was tested. Due to the oligotrophic trait of these bacteria [3], several complex cultivation media were tested for the inoculum development. It was observed that the 5-times diluted tryptone soya broth medium CM0129 by Oxoid is the most suitable medium. The PHA production was tested in subsequent cultivation in the mineral salt medium with 20 g/L of carbon substrate (glycerol or glucose). In the case of *R. spartanus*, the highest biomass concentration—2.27 g/L of cell dry mass (CDM) and P3HB content—49.52 *w/w*% of CDM were obtained on glycerol. Oppositely, *R. xylanophilus* preferred glucose utilization which resulted in a CDM value of 3.35 g/L and a P3HB content in biomass of 51.53 *w/w*%. These results confirmed that both tested *Rubrobacter* strains are capable of PHA accumulation and that the PHA content in biomass can reach high values of about 50 *w/w*% of the CDM.

**Figure 1:**
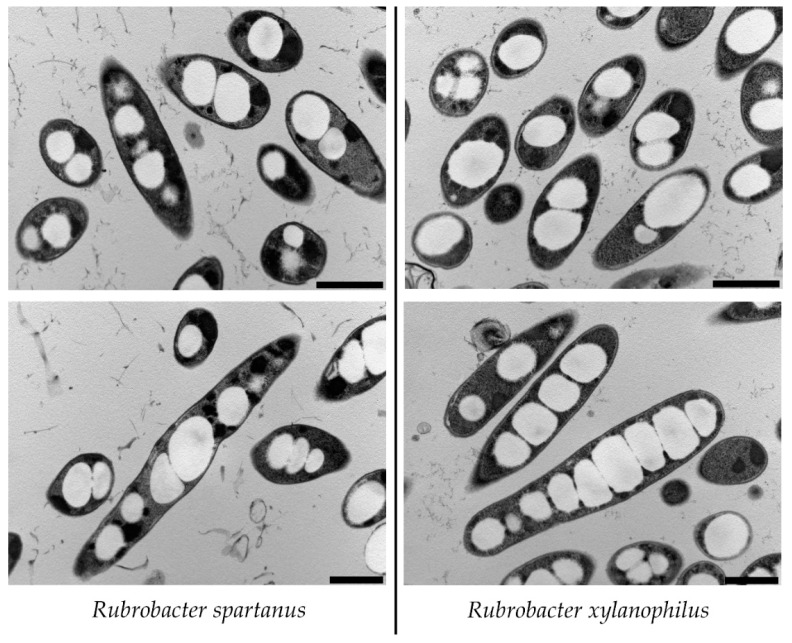
Morphology of bacterial strains *R. spartanus* (**left**) and *R. xylanophilus* (**right**) as seen by transmission electron microscopy, scale 1 μm.

The occurrence of P3HB granules in the cells, and thus the confirmation of the ability of selected species to produce P3HB, was also proved by analysis using Transmission Electron Microscopy (TEM) (Figure 1). TEM images can distort the shape of the cells as well as the distribution of the granules, as it depends on how the section is made through the cell suspension and the individual cells can be oriented differently. However, it was shown that the cultures of both strains consisted of mostly short rods containing several (2–10) PHA granules per cell, the diameter of the PHA granules was approximately 200–400 nm. It can be concluded that both observed *Rubrobacter* strains reveal the intracellular architecture, distribution, and size of PHA granules common for most described PHA producers such as *Cupriavidus necator* [30].

### 3.3. PHA Production on Various Substrates

The ability to produce PHA by *R. xylanophilus* and *R. spartanus* on various carbon sources including saccharides, glycerol, or waste frying oil (WFO) was tested and the results are shown in Table 3.

The strain *R. xylanophilus* was able to utilize all the tested substrates. A significantly higher biomass growth and P3HB titers were observed for glucose, lactose, and sucrose, the P3HB content in biomass on these substrates approached or even exceeded 50 *w/w* per CDM. The highest P3HB titers were obtained using glucose as a substrate (1.73 g/L), so this saccharide was used in the subsequent cultivations of *R. xylanophilus* as the carbon substrate. For the other carbon sources, the concentration of biomass was low and the P3HB content did not exceed 8% of the CDM, with the only exception of glycerol in which the P3HB content was approximately 38%.

Based on the available literature [3], we expected the ability of *R. spartanus* to utilize almost all selected carbon sources. Unfortunately, we were unable to confirm this assumption. Detectable bacterial growth was observed only on glucose, glycerol, and WFO. A PHA accumulation was also observed on these substrates, but the amount of synthesized P3HB was almost negligible in WFO (only 1.34 *w/w*% of the CDM). Although the highest increase in biomass was recorded when cultivated on glucose (2.70 g/L, 38.59% P3HB of the CDM), the highest proportion of P3HB was observed on glycerol (49.65% P3HB of the CDM). Because of these findings, glycerol was selected as a substrate for the subsequent cultivations of *R. spartanus*.

### 3.4. The Effect of Cultivation Temperature on PHA Accumulation

Furthermore, the effect of temperature on the growth of bacteria and their ability to accumulate PHA was investigated. As already mentioned, *R. xylanophilus* was cultivated on glucose, while *R. spartanus* on glycerol, both with constant shaking for 72 h at various temperatures ranging from 45 to 60 °C. This temperature range was chosen based on previous studies [3,21], where it was confirmed that the bacteria should grow under these conditions.

**Figure 2:**
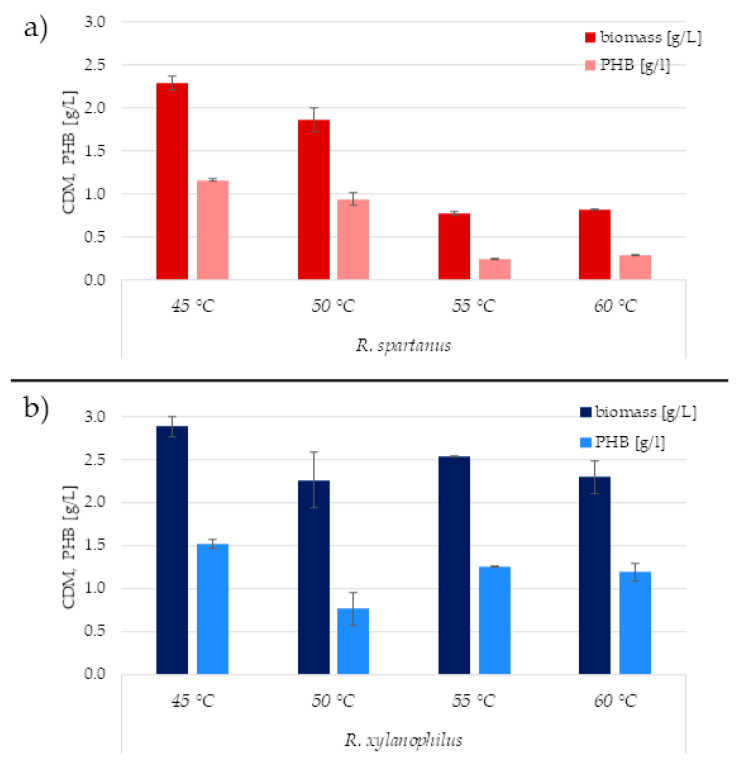
Effect of cultivation temperature on biomass growth and PHA production by (**a**) *R. spartanus* and (**b**) *R. xylanophilus*.

*R. spartanus* shows an inhibition tendency of both cell growth and PHA production with increasing temperature (Figure 2a). At 55 °C, a significant drop in bacterial growth is evident. Therefore, the optimal temperature for biopolymer production by this bacterium appears to be 45 °C. At this temperature, the biomass reached almost 2.5 g/L, with a P3HB content of approximately 50% *w/w* of the CDM.

On the other hand, *R. xylanophilus* demonstrated substantially higher robustness concerning the cultivation temperature, the chosen cultivation temperature had a rather minor effect on the P3HB accumulation since apart from the inexplicable drop in cultures cultivated at 50 °C, the P3HB content in biomass in all cultivations was about 50% *w/w* of the CDM and P3HB titers were in the interval of 1.19–1.52 g/L (Figure 2b). This confirmed our literature-based assumption that this *R. xylanophilus* reveals a truly thermophilic feature and can grow and accumulate PHA in a wide temperature range.

### 3.5. Production of PHA Copolymers

Subsequently, the ability to incorporate various monomer units into PHA polymer chains was also examined. The results are shown in Table 4. Copolymers usually have better mechanical properties than pure P3HB, which results in advantages in their eventual processing and applications [13]. For this reason, the capability of producing copolymers for production strains is very important and it depends on the available metabolic apparatus of the culture and predominantly on the substrate specificity of the PHA synthase. The precursors of 3-hydroxyvalerate (3HV), 4-hydroxybutyrate (4HB), and medium-chain length PHA (mcl-PHA) were tested. In the case of 3HV precursors, n-propanol, valeric acid, levulinic acid, n-amyl alcohol, and sodium propionate were investigated and added to the production medium at a concentration of 2 g/L. 1,6-hexanediol, 1,4-butanediol, γ-butyrolactone, and ε-caprolactone were used as 4HB precursors and sole carbon sources (8 g/L). Precursors for the mcl-PHA production were hexanoate and octanoate. These were applied to the medium at a concentration of 2 g/L. The results are demonstrated in Table 4.

Both examined strains were able to incorporate 3HV into the polymer structure when supplemented by proper structural precursors. The best total polymer yields were obtained using alcohols (n-propanol, n-amyl alcohol), however, the portion of 3HV in the copolymers is not very high. From this point of view, the use of valeric acid was more successful. In contrast, in the bacterial strain *R. spartanus*, this acid caused almost undetectable cell growth. *R. spartanus* grew well in the presence of n-propanol and sodium propionate. In the presence of n-amyl alcohol, this bacterium was able to produce a polymer consisting almost exclusively of 3HV, which is a unique feature among the natural PHA producers. On the other hand, when the bacteria were cultivated in the presence of structural precursors of 4HB or mcl-PHA, the cultures were unable to incorporate these precursors into the polymer chain since only a P3HB homopolymer was produced, hence, these data are not shown.

## 4. Discussion

The PHA accumulation capability is widely spread among numerous prokaryotes, which confirms the fact that PHA synthesis provides an important advantage for microbial cells. There are some reports that also members of the actinobacteria phylum are capable of PHA synthesis. Matias et al. screened the PHA synthesis potential in a large number of actinomycetes isolated from soil in the Rio Grande do Sul location in Brazil and they observed that 44% of the isolates were Sudan Black and Nile red staining positive which indicates their capability for PHA production [31]. Actinobacteria were also identified as an important group of PHA-producing organisms isolated from the Pangi-Chamba trans-Himalayan region [32]. Some PHA accumulating actinomycetes, especially members of the *Rhodococcus* genus, are even considered being interesting strains for biotechnological production of PHA [33,34]. Our results demonstrate and prove that the PHA biosynthesis capacity is also widely spread among members of the genus *Rubrobacter,* belonging to *Actinobacteria* phylum. We identified the presence of *phaC* genes encoding for PHA synthase in all *Rubrobacter* species whose complete genomes are available in public databases (see Table 1). Interestingly, all strains contain genes encoding for two different PHA synthases. Firstly, there are genes encoding for class I PHA synthase, an enzyme composed only of a PhaC subunit which catalyzes the synthesis of PHA with monomer units comprising 3–5 carbon atoms (scl-PHA). Secondly, there are also operons encoding PhaC and PhaE subunits of class III PHA synthase, they are also specific for the production of scl-PHA, but some class III PHA synthases reveal relatively low substrate specificity and can incorporate also medium-chain-length monomers (six and more carbon atoms) into the PHA structure [35]. Identification of phaE subunits is problematic as all identified *phaE* genes within the genus *Rubrobacter* have similar homologies to publicly available *phaE* as well as *phaR* genes. The PhaR subunit can be found in class IV PHA synthases which also synthetize scl-PHA. While the average molecular weight of PhaR is 20 kDa, PhaE can have 20 or 40 kDa [36]. As the predicted molecular weight of the identified PhaE subunits is ~35 kDa, we believe that the identified PHA synthases truly belong to class III. Apart from the bioinformatics analysis, we have also experimentally confirmed PHA biosynthesis on the phenotype level in two representatives of the genus—*R. xylanophilus* and *R. spartanus*, both strains revealed common morphology and distribution of PHA granules in bacterial cells (see Figure 1) and the PHA content in bacterial cells can reach high values of about 50% of the CDM (Table 3). Furthermore, apart from PHA synthases, we also identified several extracellular PHA depolymerases (see Table 2) which indicates that some *Rubrobacter* species can be involved in the biological decomposition of PHA polymeric materials in the environment.

The fact that multiple *phaC* genes are present in numerous *Rubrobacter* species and cellular PHA content achieves high values underlines the importance of PHA for the *Rubrobacter* genus. The presence of PHA granules in bacteria has very important consequences concerning the fitness and robustness of bacteria when exposed to various stress conditions [37]. Slaninova et al. described that PHA granules are capable of efficient scattering of UV irradiation, which protects DNA, the most UV-radiation sensitive molecule, from damage and also reduces the number of free radicals present in radiation-exposed cells [17]. Therefore, it seems likely that in the radiation-tolerant *Rubrobacter* species the PHA granules might also serve as a radiation protectant in addition to pigments and robust enzyme machinery. Furthermore, PHA granules are also able to protect bacterial cells from losing cellular integrity when exposed to hypertonic conditions [23] or fluctuations in external osmolarity [14]. Since the PHA accumulation is widely spread among halophiles including the extremely halophilic archaea such as *Haloferax mediterranei*, it was recently suggested that the PHA accumulation could be one of the possible strategies for adaptation of microorganisms to high salinity environments [38]. So along with the accumulation of compatible solutes, the PHA can also contribute to the halotolerant feature of the *Rubrobacter* species. The PHA metabolism is sometimes called a PHA cycle since PHA are simultaneously synthesized and degraded in bacterial cells, which results in an increased intracellular pool of PHA monomers. From a stress robustness point of view, this is also of high importance since PHA monomers such as 3-hydroxybutyrate are potent chemical chaperones protecting biomolecules from denaturation by heating or oxidative pressure [39,40] and also reveal substantial cryoprotective activity [16]. Additionally, phasins (PhaP), PHA granules-associated proteins, reveal a chaperoning activity and protect proteins from denaturation [41]. These facts can also contribute to the thermotolerance or even thermophilic trait of the *Rubrobacter* species. Therefore, it seems that PHA might be important metabolites with respect to the multiple extremophilic attributes of the *Rubrobacter* genus members and, therefore, PHA could be classified as extremolytes.

PHA accumulation in *Rubrobacter* species is not only of fundamental importance but might be also interesting from a biotechnological point of view. Currently, mostly Gram-negative bacteria, such as *Cupriavidus necator* [42], *Burkholderia sacchari* [43], or various *Halomonas* species [44] are preferentially employed for PHA production. Nevertheless, pyrogenic lipopolysaccharides present in the outer membranes of Gram-negative bacteria are co-isolated along with PHA and represent serious contamination of the material, which complicates the utilization of PHA in numerous fields such as medicine, cosmetics or food packaging [45]. Moreover, the Gram-positive *Bacillus* and related species can be used for PHA synthesis [46] which overcome the problems with PHA materials contamination by pyrogens. Nevertheless, bacilli are spore-forming bacteria which bring numerous complications to the process since sudden sporulation stops the desirable metabolic activity of the culture and is associated with the mobilization of PHA storage which reduces product titers and yields [47]. From this perspective, the Gram-positive non-sporulating *Rubrobacter* species seems to be an interesting option.

Another reason why *Rubrobacter* spp. could be of biotechnological interest is their multi-extremophilic feature which enables their utilization within the concept of Next-generation Industrial Biotechnology. Extreme conditions prevent the process from microbial contamination, which puts low demands on sterilization requirements and enables the operation of the process in a more efficient continuous or semi-continuous mode [12]. Therefore, we focused on the study of PHA production by two thermophilic members of the *Rubrobacter* genus. Especially *R. xylanophilus* could be interesting in this context since it is capable of growth and PHA production even at 60 °C (see Figure 2). The employment of thermophiles could seem to be energetically demanding since heating is required, nevertheless, due to the metabolic heat generated by the culture and energy dissipated by the stirrer, the process can be theoretically operated as (at least partially) a self-heating system. Moreover, no energetically demanding cooling is required. Therefore, processes employing thermophiles can be surprisingly energetically feasible [48]. Both tested strains are capable of accumulation of substantial amounts of PHA on simple and cheap mineral media, however, *R. xylanophilu*s seems to be the more flexible strain in terms of a range of utilizable substrates. Apart from the preferred glucose, reasonable amounts of PHA were obtained even with sucrose or lactose (Table 3), which indicates the potential of the culture to be used for PHA production from molasses or dairy industry wastes such as cheese whey. It should be stated that the process of PHA production and cultivation conditions were not optimized in this work, therefore, it is likely that by precisely setting the conditions, the obtained product titers, as well as yields, could be substantially improved. What could be an important obstacle is the oligotrophic characteristic of the culture, which might complicate obtaining high cell density, an important prerequisite of a feasible PHA production. Nevertheless, the main aim of this study was the description of the PHA synthetic capability and potential of the selected *Rubrobacter* species. A detailed optimization of the PHA production and an uncovering of production limits of the cultures will be performed in follow-up studies.

When evaluating the suitability of the particular culture as a candidate for PHA production, apart from product titers and yields, it is also necessary to take into account its capability of incorporation of various monomers into the polymer structure. The P3HB homopolymer is, due to its high crystallinity, a brittle and stiff material with very limited flexibility. Moreover, its melting temperature of about 170 °C is very close to the degradation temperature (about 200 °C), which complicates its processing. However, a supplementation of the culture with appropriate structural precursors can result in the incorporation of other monomer units such as 3HV or 4HB into the polymer chain, which substantially improves its mechanical as well technological properties [13]. As shown in Table 1, *R. xylanophilus* DSM 9941 possesses two PHA synthases belonging to class I and class III. Since the same combination was detected for additional *Rubrobacter* species and unidentified strains, it is probable that *R. spartanus* DSM 102139 carries the same enzymes. There are reports that especially PHA synthases belonging to class III are capable of incorporation of various monomers into the polymer chain. For instance, *Cupriavidus necator* harboring broad-specificity PHA synthase of *Rhodococcus aetheriovarans* is capable of accumulation of a copolymer containing 3-hydroxybutyrate and 3-hydroxyhexanoate [49]. Nevertheless, the *Rubrobacter* strains tested in this work were capable only of incorporation of 3-hydroxyvalerate subunits when supplemented with precursors containing an odd number of carbon atoms. Interesting is a very high portion of 3-hydroxyvalerate in the polymer produced by *R. spartanus* in the presence of n-amyl alcohol or propionate (see Table 4). From a fundamental point of view, it would be interesting to look into the substrate specificity of both PHA synthases (class I and class III) and into their involvement in the PHA biosynthesis in both *Rubrobacter* strains which will be also the goal of future experiments.

## 5. Conclusions

Our results indicate that the PHA biosynthesis capability is a common feature among the members of the *Rubrobacter* genus. Since the PHA metabolism and even the simple presence of PHA granules in cells substantially enhances the stress robustness of bacteria, it is very likely that, even in the *Rubrobacter* spp., PHA contribute to their astonishing stress tolerance and durability. Furthermore, since the *Rubrobacter* species are Gram-positive, non-sporulating, capable of the utilization of numerous substrates and they also reveal multi-extremophilic features, they can be considered as interesting candidates for PHA production within the concept of Next-generation Industrial Biotechnology.

## Figures and Tables

**Table 1:** Overview of PHA synthases in *Rubrobacter* species whose complete genomes are available in public databases.

Organism	Accession No.	Putative PHA Class	Gene	Locus
*Rubrobacter* sp. SCSIO 52909	CP045119.1	Class I	*phaC*	GBA63_04560
		Class III	*phaC*	GBA63_04605
			*phaE*	GBA63_04610
*Rubrobacter* sp. SCSIO 52915	CP045121.1	Class I	*phaC*	GBA65_16640
		Class III	*phaC*	GBA65_16620
			*phaE*	GBA65_16615
*R. xylanophilus* AA3-22	AP019791.1	Class I	*phaC*	RxyAA322_15910
		Class III	*phaC*	RxyAA322_15900
			*phaE*	RxyAA322_15890
*R. xylanophilus* DSM 9941	CP000386.1	Class I	*phaC*	Rxyl_2386
		Class III	*phaC*	Rxyl_2385
			*phaE*	Rxyl_2384
*R. radiotolerans* RSPS-4	CP007514.1	Class I	*phaC*	RradSPS_2088
		Class I	*phaC*	RradSPS_2601
		Class III	*phaC*	RradSPS_2085
			*phaE*	RradSPS_2084
*R. indioceani* SCSIO 08198	NZ_CP031115.1	Class I	*phaC*	DU509_RS10855
		Class I	*phaC*	DU509_RS11050
		Class III	*phaC*	DU509_RS10845
			*phaE*	DU509_RS15705

**Table 2:** Overview of extracellular dPHAscl depolymerases identified in members of the *Rubrobacter* genus.

Organism	Accession
*Rubrobacter* sp. MGR_bin435	MBA3953681.1
*Rubrobacter* sp. Co-bin5	MBD0252984.1
*Rubrobacter radiotolerans* RSPS-4	AHY46637.1
*Rubrobacter radiotolerans* DSM 5868	SMC05048.1
*Rubrobacter* sp. SCSIO 52909	QIN83803.1
*Rubrobacter* sp. MGR_bin143	MBA2690884.1
*Rubrobacter* sp. MGR_bin332	MBA3609848.1
*Rubrobacter indicoceani* SCSIO 08198	WP_119067871.1
*Rubrobacter* sp. MGR_bin27	MBA3471765.1
*Rubrobacter* sp. SCSIO 52915	QIN79014.1
*Rubrobacter* sp. MGR_bin77	MBA3388321.1
*Rubrobacter* sp. MGR_bin253	MBA2376553.1
*Rubrobacter* sp. MGR_bin199	MBA2534341.1
*Rubrobacter* sp. MGR_bin231	MBA2443425.1
*Rubrobacter* sp. MGR_bin80	MBA3425602.1

**Table 3:** The ability to utilize various carbon substrates for growth and PHA synthesis by bacteria *R. xylanophilus* (R1) and *R. spartanus* (R2)**.**

	Substrate	CDM ^1^ [g/L]	PHB [% of CDM]	P3HB [g/L]
**R1**	arabinose	0.47 ± 0.16	3.00 ± 0.04	0.01 ± 0.01
	fructose	0.82 ± 0.11	4.21 ± 0.16	0.03 ± 0.01
	galactose	0.73 ± 0.10	7.28 ± 0.20	0.05 ± 0.01
	glucose	3.35 ± 0.03	51.53 ± 0.30	1.73 ± 0.07
	glycerol	0.92 ± 0.06	38.02 ± 0.13	0.35 ± 0.01
	lactose	2.38 ± 0.06	52.28 ± 0.14	1.24 ± 0.01
	mannose	0.96 ± 0.04	4.56 ± 0.19	0.04 ± 0.01
	WFO	0.49 ± 0.17	2.34 ± 0.05	0.01 ± 0.01
	succrose	2.10 ± 0.04	45.28 ± 8.41	0.95 ± 0.18
	xylose	1.46 ± 0.27	4.39 ± 0.23	0.06 ± 0.01
	**Substrate**	**CDM [g/L]**	**PHB [% of CDM]**	**P3HB [g/L]**
**R2**	arabinose	n. d. ^2^	n. d.	n. d.
	fructose	n. d.	n. d.	n. d.
	galactose	n. d.	n. d.	n. d.
	glucose	2.70 ± 0.04	38.59 ± 2.84	1.04 ± 0.08
	glycerol	2.27 ± 0.07	49.65 ± 0.18	1.13 ± 0.01
	lactose	n. d.	n. d.	n. d.
	mannose	n. d.	n. d.	n. d.
	WFO	2.50 ± 0.01	1.34 ± 0.05	0.03 ± 0.00
	succrose	n. d.	n. d.	n. d.
	xylose	n. d.	n. d.	n. d.

^1^ CDM—cell dry mass, ^2^ n.d.—not detected, results are presented in the form mean ± standard deviation.

**Table 4:** Ability to produce copolymers containing 3HV from different types of precursors, R1—*R. xylanophilus,* R2—*R. spartanus* (72 h, 45 °C, 180 rpm)**.**

		*n*-Propanol	Valeric Acid	Levulinic Acid	*n*-Amyl Alcohol	Propionate
**R1**	CDM [g/L]	2.37 ± 0.01	0.85 ± 0.01	n.d.*	2.15 ± 0.08	0.58 ± 0.01
	PHA [% of CDM]	31.38 ± 0.06	3.57 ± 0.03	n.d.	30.73 ± 1.31	4.63 ± 0.04
	PHA [g/L]	0.74 ± 0.01	0.03 ± 0.01	n.d.	0.66 ± 0.03	0.03 ± 0.01
	3HV [mol. %]	4.27	8.42	n.d.	2.78	0.00
	3HB [mol. %]	95.73	91.58	n.d.	97.22	100.00
**R2**	CDM [g/L]	2.23 ± 0.01	n.d.	0.45 ± 0.01	1.29 ± 0.04	2.08 ± 0.02
	PHA [% of CDM]	43.86 ± 1.42	n.d.	13.26 ± 0.03	32.73 ± 0.73	20.62 ± 0.43
	PHA [g/L]	0.98 ± 0.03	n.d.	0.06 ± 0.01	0.42 ± 0.02	0.43 ± 0.01
	3HV [mol. %]	0.77	n.d.	0.00	99.48	87.04
	3HB [mol. %]	99.23	n.d.	100.00	0.52	12.96

* n.d.—not detected, results are presented in the form mean ± standard deviation.
